# Approaches to Human Health Risk Assessment Based on the Signal-To-Noise Crossover Dose

**DOI:** 10.1289/ehp.1205212

**Published:** 2012-07-02

**Authors:** Weihsueh A. Chiu, Kathryn Z. Guyton, Karen Hogan, Jennifer Jinot

**Affiliations:** National Center for Environmental Assessment, Office of Research and Development, U.S. Environmental Protection Agency, Washington, DC, E-mail: chiu.weihsueh@epa.gov

We acknowledge the effort of Sand et al. (2011) in striving to develop a transparent, objective procedure for point of departure (POD) estimation, as encouraged by scien-tific review groups (National Research Council 2009). Although additional charac-teri-za-tion of the statistical properties of the signal-to-noise crossover dose (SNCD) may be warranted, the goal of Sand et al. (2011) appears consistent with the intent of the POD to charac-terize “the beginning of extrapolation to lower doses” [U.S. Environmental Protection Agency (EPA) 2005]. In this letter we respond to the authors’ illustration of their approach using cancer bio-assay data to develop reference doses (RfDs) that target a 1/1,000 risk through linear extrapolation from the POD by highlighting opportunities to augment their statistically based approach with biological considerations.

For most carcinogens, the U.S. EPA develops cancer potency estimates as follows (U.S. EPA 2005). A POD associated with a benchmark response level (BMR) is derived and converted to human-equivalent units (incorporating information about cross-species dose scaling). The BMR is then divided by the human-equivalent POD to obtain a potency estimate, under the assumption that risks extrapolate linearly with doses below the BMR. For Sand et al. (2011), the upper-bound extra risk estimate (UER_SNCD_) is the BMR associated with the SNCD, but we recom-mend expressing SNCDs in human equivalents before deriving potency estimates.

For nonlinear extrapolation resulting in a RfD (which the U.S. EPA uses for non-cancer effects and carcinogens with a threshold mode of action), Sand et al. (2011) chose to linearly extrapolate to a 1/1,000 risk in the test animal, which they considered analogous to applying a 100-fold uncertainty factor to a BMDL_10_ (lower bound on the benchmark dose corresponding to 10% extra risk). Several aspects of this proposal merit further considera-tion. First, margins of exposure much larger than 100-fold would be typical for cancer. Furthermore, whereas linear extrapo-la-tion involves extrapolation in the same population to a smaller level of effect, the standard uncertainty factor approach involves extrapolation across populations at a fixed level of effect. The alternative we propose separately accounts for these biologically unrelated processes.

Motivating our proposal is the need highlighted by Sand et al. (2011) to clearly separate statistical factors supporting the level of effect associated with the POD while also fully incorporating biological considerations. We propose specifying “target” effect levels (TELs) associated with different end points based on biological considerations, independent of data set. The TELs could then be compared with the lowest practical BMR for a given data set—the UER_SNCD_ used by Sand et al. The UER_SNCD_/TEL ratio is a diagnostic of the extent of extrapolation to the TEL. If UER_SNCD_ ≤ TEL, then the BMD at the TEL does not involve extrapo-la-tion and can serve as the POD. For a UER_SNCD_ > TEL, the greater the ratio, the greater the uncertainty in the BMD at the TEL from extrapo-la-tion below the SNCD. In this case, the SNCD could serve as the POD, and the gap between the UER_SNCD_ and the TEL could be bridged by an additional factor (analo-gous to the LOAEL-to-NOAEL factor) or linear extrapolation. Then, inter-species, intra-species, and any other adjustments for deriving RfDs would be applied as usual. Thus, this approach separately takes into account biological considerations related to the severity of the end point (via the TEL), statistical considerations related to the study data (via the UER_SNCD_), and adjustments from the test species to sensitive humans (via uncertainty factors or chemical-specific adjustments).

In sum, the work of Sand et al. (2011)advances the development of approaches for providing a transparent, objective method to demark where “extrapolation begins.” However, for human health risk assessment, we propose augmenting statistically based approaches so that inter- and intra-species adjustments and biological considerations relating to the end points are explicitly addressed. Although consensus on specifying TELs may be challenging, particularly for precursor or toxico-genomic end points, clearly separating biological and statistical considerations will enhance the transparency and consistency of chemical assessments.
